# Sulindac metabolites inhibit epidermal growth factor receptor activation and expression

**DOI:** 10.1186/1477-3163-4-16

**Published:** 2005-09-02

**Authors:** Heather A Pangburn, Hanna Kraus, Dennis J Ahnen, Pamela L Rice

**Affiliations:** 1 Molecular Toxicology and Environmental Health Sciences Program, Department of Pharmaceutical Sciences, University of Colorado Health Sciences Center, Denver, USA; 2 Veterans Administration Medical Center, Denver, USA; 3 Department of Medicine, University of Colorado Health Sciences Center, Denver, USA; 4 University of Colorado Comprehensive Cancer Center, Denver, USA

## Abstract

**Background:**

Regular use of nonsteroidal anti-inflammatory drugs (NSAIDs) is associated with a decreased mortality from colorectal cancer (CRC). NSAIDs induce apoptotic cell death in colon cancer cells *in vitro *and inhibit growth of neoplastic colonic mucosa *in vivo *however, the biochemical mechanisms required for these growth inhibitory effects are not well defined. We previously reported that metabolites of the NSAID sulindac downregulate extracellular-signal regulated kinase 1/2 (ERK1/2) signaling and that this effect is both necessary and sufficient for the apoptotic effects of these drugs. The goal of this project was to specifically test the hypothesis that sulindac metabolites block activation and/or expression of the epidermal growth factor (EGF) receptor (EGFR).

**Methods:**

HT29 human colon cancer cells were treated with EGF, alone, or in the presence of sulindac sulfide or sulindac sulfone. Cells lysates were assayed by immunoblotting for phosphorylated EGFR (pEGFR, pY1068), total EGFR, phosphorylated ERK1/2 (pERK1/2), total ERK1/2, activated caspase-3, and α-tubulin.

**Results:**

EGF treatment rapidly induced phosphorylation of both EGFR and ERK1/2 in HT29 colon cancer cells. Pretreatment with sulindac metabolites for 24 h blocked EGF-induced phosphorylation of both EGFR and ERK1/2 and decreased total EGFR protein expression. Under basal conditions, downregulation of pEGFR and total EGFR was detected as early as 12 h following sulindac sulfide treatment and persisted through at least 48 h. Sulindac sulfone induced downregulation of pEGFR and total EGFR was detected as early as 1 h and 24 h, respectively, following drug treatment, and persisted through at least 72 h. EGFR downregulation by sulindac metabolites was observed in three different CRC cell lines, occurred prior to the observed downregulation of pERK1/2 and induction of apoptosis by these drugs, and was not dependent of caspase activation.

**Conclusion:**

These results suggest that downregulation of EGFR signaling by sulindac metabolites may occur, at least in part, by inhibiting activation and expression of EGFR. Inhibition of EGFR signaling may account for part of the growth inhibitory and chemopreventive effects of these compounds.

## Background

CRC is the second most common cause of cancer death in the USA, with an estimated annual incidence of 104,950 and mortality of 56,290 in 2005 [1]. The lifetime risk of developing CRC in the general US population is almost 6% [1]. Effective preventive measures could substantially reduce both the incidence and mortality from CRC. NSAIDs are one of the most widely studied and promising groups of compounds for CRC prevention.

NSAIDs mediate their anti-inflammatory effects by inhibiting the enzymatic activity of cyclooxygenase-1 (COX-1) and/or COX-2. Sulindac is a non-selective NSAID that inhibits both COX-1 and COX-2. Sulindac is rapidly metabolized in the liver to two major metabolites; 1) sulindac sulfide, which is an active NSAID, and 2) sulindac sulfone, which does not inhibit COX enzymatic activities and thus is not an NSAID. A large body of evidence from cell culture, animal model, human epidemiologic and clinical studies indicates that NSAIDs, including sulindac and aspirin, have potent chemopreventive and chemoregressive properties against colon cancer [2,3]. Although substantial evidence indicates that NSAIDs inhibit the growth of neoplastic colonic mucosa, the biological and biochemical mechanisms responsible for the growth inhibitory effects of these drugs are not well defined. The chemopreventive and chemoregressive effects of NSAIDs may not be due to inhibition of COX alone as we have shown that sulindac sulfide and sulindac sulfone both inhibit the phosphorylation of ERK1/2 in HCT15 cells that do not express COX-1 or COX-2 [4]. Additionally, sulindac sulfone, the non-NSAID metabolite of sulindac, has been shown, by our lab and others, to induce apoptosis of cancer cells *in vitro*, prevent tumor formation in animal models, and cause regression of adenomas in familial adenomatous polyposis (FAP) [2,5].

Several biological mechanisms for the chemopreventive effects of NSAIDs have been proposed including inhibition of proliferation, induction of apoptosis, and inhibition of angiogenesis. Our laboratory [5,6] and others [2] have reported that NSAIDs inhibit growth of CRC cells in culture primarily by the induction of apoptotic cell death. An apoptotic mechanism was also suggested in human adenomas treated with sulindac sulfone [7]. We have reported that the apoptotic effect of sulindac is not dependent on COX inhibition [4,5,8], but does appear to be dependent on the downregulation of ERK1/2 [8].

Work from several laboratories has demonstrated substantial interactions between the biochemical effects of NSAIDs and EGFR signaling. It is well established that COX-2 protein expression is stimulated by EGF and decreased by EGFR inhibitors [9]. Sulindac sulfide and indomethacin inhibit TGFα induced prostaglandin production and thymidine incorporation in RIE-1 cells [10], and indomethacin, ibuprofen, and aspirin all block EGF-induced Ca^++ ^influx in Caco-2 colon cancer cells [11]. Furthermore, combination therapy of NSAIDs and EGFR antagonists display an additive effect against colon tumor development *in vivo *[12] and two previous studies suggest that NSAIDs might inhibit EGFR signaling [13,14]. Finally, sulindac has been shown to inhibit expression of ErbB2/HER2 protein expression in rectal mucosa of FAP patients [15]. The biochemical mechanisms by which NSAIDs might modulate EGFR signaling has not been addressed in any of these studies.

**Figure 1:**
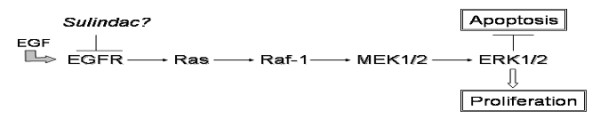
**The EGFR/MAPK/ERK1/2 signaling pathway**. We have previously shown that sulindac metabolites inhibit both MEK1/2 and ERK1/2 activation. The goal of this study was to determine if this inhibition is due to downregulation of the EGFR.

We have previously shown that sulindac sulfide and sulindac sulfone inhibit ERK1/2 phosphorylation both basally and in response to EGF [6], and that this inhibition is sufficient [5] and necessary [8] for the apoptotic effect of these drugs. The goal of this study was to specifically test the hypothesis that sulindac metabolites block activation and/or expression of EGFR (Figure 1). We report, for the first time, that sulindac metabolites block both basal and EGF induced phosphorylation of EGFR and downregulate total EGFR protein expression. Together these results identify a new target for the actions of sulindac and suggest that inhibition of EGFR activation may be a major biochemical mechanism for both the downregulation of ERK1/2 signaling and the chemopreventive activity of NSAIDs.

## Methods

### Reagents

Cell culture media and fetal bovine serum (FBS) were purchased from Mediatech (Herndon, VA), antibiotic/antimycotic solution (penicillin/streptomycin/fungizone) from Life Technologies, Inc. (Grand Island, NY), and tissue culture plates from Falcon (Franklin Lakes, NJ). Primary antibodies raised against phosphorylated ERK1/2 (Thr202/Tyr204), total ERK1/2, and total EGFR were purchased from Santa Cruz Biotechnology (Santa Cruz, CA); primary antibody against phosphorylated EGFR(Tyr1068), horseradish peroxidase-conjugated anti-mouse and anti-rabbit secondary antibodies, and EGF were purchased from Biosource (Camarillo, CA). Primary antibody against caspase-3 (recognizes both full length caspase-3 and cleavage products) and cleaved caspase-3 (recognizes only cleavage products of activated caspase-3 resulting from cleavage adjacent to Asp175) were obtained from Cell Signaling Technology (Beverly, MA). Immobilon-P membranes were purchased from Millipore (Bedford, MA), chemiluminescent visualization reagents from NEN (Boston, MA), and X-ray film from Pierce (Rockford, IL). Sulindac sulfide was from LKT Laboratories Inc. (St. Paul, MN). ZVAD-fmk (caspase inhibitor I) and primary antibody against α-tubulin were purchased from Calbiochem (San Diego, CA). Sulindac sulfone was a generous gift from Cell Pathways Inc. (Horsham, PA).

### Cell culture

HT29, Caco-2, and HCT116 human colon cancer cells were purchased from American Type Culture Collection (Manassas, VA). HT29 and HCT116 cells were maintained in RPMI 1640 media supplemented with 10% FBS and 1% penicillin/streptomycin/fungizone solution; Caco-2 cells were maintained in DMEM media with 4 mM L-glutamine adjusted to contain 1.5 g/L glucose and supplemented with 0.01 mg/ml human transferring, 10% FBS, and 1% penicillin/streptomycin/fungizone solution. Cells were plated and grown on tissue culture plates as specified in the results section.

### Morphologic quantitation of apoptotic cell death

An ethidium bromide/acridine orange double-dye morphological assay [16] was used to quantitate apoptotic cell death induced by sulindac metabolites. Cells were grown in multi-well plates and given the appropriate drug treatment. Three days after treatment, cells were harvested and stained with a mixture of 100 μg/ml ethidium bromide and 100 μg/ml acridine orange and then examined by fluorescence microscopy. Three fields of 100 cells each were counted and the number of apoptotic cells (as determined by nuclear morphology) as well as the number of living and necrotic cells were expressed as a percentage of total cells counted.

### Cell lysates and Western immunoblotting

At time of harvest, cells were scraped from the plates, washed with ice-cold phosphate buffered saline (PBS), and pelleted at 2,400 × g for 5 min. Care was taken to keep cells as cold as possible during the entire procedure. After aspirating the supernatant, the cells were washed twice with ice cold PBS and centrifuged. The cell pellet was lysed in cell extraction buffer (10 mM Tris, pH 7.4, 100 mM NaCl, 1 mM EDTA, 1 mM EGTA, 1 mM NaF, 20 mM Na_4_P_2_O_7_, 2 mM Na_3_VO_4_, 1% Triton X-100, 10% glycerol, 0.1% SDS, 0.5% deoxycholate, 1 mM PMSF, 60 μg/mL aprotinin, 10 μg/mL leupeptin, 1 μg/mL pepstatin) for 30 min, on ice, with vortexing at 10 min intervals. Lysates were then centrifuged at 18,000 × g for 10 min at 4°C, and supernatants collected. Protein concentrations of lysates were determined by the method of Lowry [17].

Lysate samples (50 μg total protein) were separated by sodium dodecyl sulfate-polyacrylamide gel electrophoresis and electrotransferred overnight onto Immobilon-P polyvinylidene difluoride membranes. Nonspecific binding was blocked by immersing membranes in Tris-neutral saline with 1% (w/v) dry milk and 0.05% Tween 20 for 30 min at room temperature, then blots were incubated with pEGFR, pERK1/2, caspase 3, cleaved caspase-3, or α-tubulin primary antibody while rocking at 37°C for 1 h. Immunoreactive protein was detected by incubating blots with horseradish peroxidase-conjugated secondary antibody for 1 h followed by chemiluminescent substrate for 1 min. Membranes probed for phospho-proteins were then stripped and reprobed with total EGFR or total ERK1/2 primary antibody followed by horseradish peroxidase-conjugated secondary antibody as described above. Non-stripped control blots were run to show that this stripping procedure gave the same results as primary blots with each antibody. Immunoreactive proteins were visualized by exposure to radiographic film. Quantitation of protein levels was determined by densitometry using a visual imaging system (UN-SCAN-IT gel, Silk Scientific, Inc., Orem, UT).

### Statistical Analysis

Data was analyzed by one way ANOVA using a Newman-Keuls multiple comparison test and statistical significance accepted at p < 0.05 (GraphPad Prism 4.0, GraphPad Software Inc., San Diego, CA).

## Results

### EGF induces phosphorylation of EGFR and ERK1/2 in HT29 human colon cancer cells

**Figure 2:**
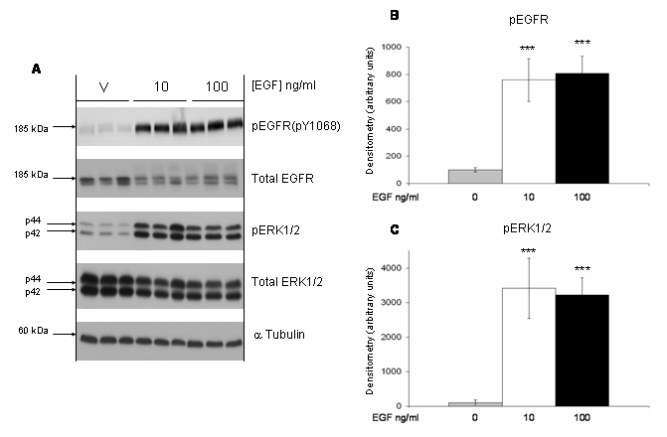
**EGF induces EGFR and ERK1/2 phosphorylation**. HT29 human colon cancer cells were grown to 80% confluence in medium containing 10% FBS then serum deprived for 48 h before treatment with vehicle (water), 10, or 100 ng/ml EGF. Cells were harvested 10 min after EGF treatment and lysates prepared for **(A) **Immunoblotting with antibodies raised against pEGFR (pY1068), total EGFR, pERK1/2, and total ERK1/2; α-tubulin immunoblots of the same lysates served as loading controls. The graphs show the densitometry results of the pEGFR bands **(B) **and pERK1/2 bands **(C) **normalized for the loading controls. Data represent mean of triplicate samples ± SD; statistical significance is denoted by ***p < 0.001 *versus *vehicle. Results shown in the figure are representative of 2 separate experiments each with triplicate samples.

In order to verify that EGFR signaling was intact in our cells, the effects of EGF treatment on the expression and activation of EGFR and ERK1/2 in HT29 human colon cancer cells was examined. It was observed that EGF rapidly induced a > 7 fold increase of pEGFR that was maximal at 10 min (Figure 2A and 2B). Similarly, EGF induced a rapid, > 30 fold, increase of pERK1/2 that was also maximal at 10 min (Figure 2A and 2C). EGF-induced phosphorylation of EGFR and ERK1/2 persisted through 60 min, and was no longer detected at 24 h (data not shown). Similar results were obtained in HCT116 and Caco-2 human colon cancer cell lines (data not shown).

### Sulindac metabolites block EGF-induced phosphorylation of EGFR and ERK1/2, downregulate total EGFR protein, and induce apoptotic cell death

**Figure 3:**
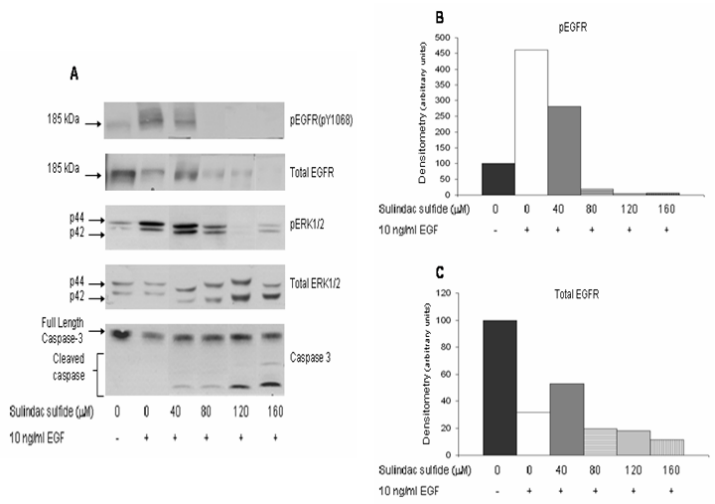
**Dose response of sulindac sulfide inhibition of EGFR**. HT29 cells were grown to 80% confluence in medium containing 10% FBS then serum deprived for 24 h before addition of drug. Cells were then treated with vehicle (0.1% DMSO), 40, 80, 120, or 160 μM sulindac sulfide, drug doses previously shown to induce apoptotic cell death in these cells. Twenty four hours after drug treatment, vehicle or 10 ng/ml EGF was added and cells were harvested 10 min later. **(A) **Immunoblots were performed on cell lysates with antibodies raised against pEGFR (pY1068), total EGFR, pERK1/2, total ERK1/2, and caspase-3; total ERK1/2 immunoblots served as loading controls. The graphs show the densitometry results of the pEGFR bands **(B) **and total EGFR bands **(C)**. Results shown in figure are representative of 3 separate experiments.

We next determined the effect of sulindac sulfide on EGF-induced phosphorylation and expression of EGFR and ERK1/2. Pretreatment of HT29 cells for 24 h with 40–160 μM sulindac sulfide blocked the ability of EGF to induce phosphorylation of EGFR in a dose dependent fashion. Total EGFR expression and EGF-induced phosphorylation of ERK1/2 proteins was also downregulated following pretreatment with sulindac sulfide (Figure 3). There was no effect of drug or EGF treatment on total ERK1/2 expression.

**Figure 4:**
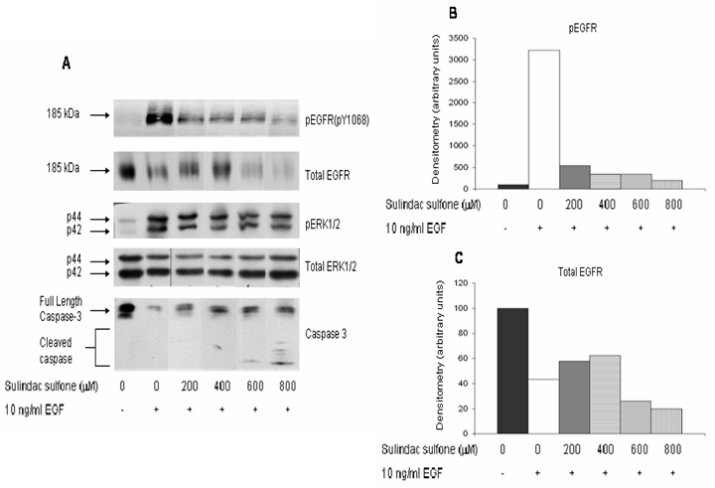
**Dose response of sulindac sulfone inhibition of EGFR**. HT29 cells were grown to 80% confluence in medium containing 10% FBS then serum deprived for 24 h before addition of drug. Cells were then treated with vehicle (0.2% DMSO), 200, 400, 600, or 800 μM sulindac sulfone, drug doses previously shown to induce apoptotic cell death in these cells. Twenty four hours after drug treatment, vehicle or 10 ng/ml EGF was added and cells were harvested 10 min later. **(A) **Immunoblots were performed on cell lysates with antibodies raised against pEGFR (pY1068), total EGFR, pERK1/2, total ERK1/2, and caspase-3; total ERK1/2 immunoblots served as loading controls. The graphs show the densitometry results of the pEGFR bands **(B) **and total EGFR bands **(C)**. Results shown in figure are representative of 3 separate experiments.

The effect of sulindac sulfone on EGF-induced phosphorylation of EGFR and ERK1/2 was also examined. As seen with sulindac sulfide, pretreatment of HT29 cells for 24 h with 200–800 μM sulindac sulfone markedly decreased the ability of EGF to induce phosphorylation of EGFR; total EGFR expression levels and ERK1/2 phosphorylation were also downregulated (Figure 4), albeit to a lesser extent than with sulindac sulfide.

The finding that total EGFR protein expression was decreased by sulindac metabolites (Figures 3 and 4) was unexpected; we therefore confirmed these results using a second antibody (Biosource catalog #AHR5062). The confirmatory EGFR antibody is an EGFR antibody cocktail (composed of four monoclonal antibodies) directed against both the extracellular and cytoplasmic domains of EGFR. The same downregulation of total EGFR elicited by sulindac sulfide and sulfone was found using this antibody (data not shown), confirming that these results are not peculiar to a single EGFR epitope or a result of an artifact of the use of a single antibody.

EGF had no effect on morphological apoptosis or activation of caspase-3 but treatment with 40–160 μM sulindac sulfide (Figure 3) or 600–800 μM sulindac sulfone (Figure 4) induced substantial apoptotic cell death, as determined by cleavage of caspase-3 and confirmed with the acridine orange and ethidium bromide morphologic assay (data not shown).

### Sulindac metabolites downregulate basal EGFR phosphorylation and expression

**Figure 5:**
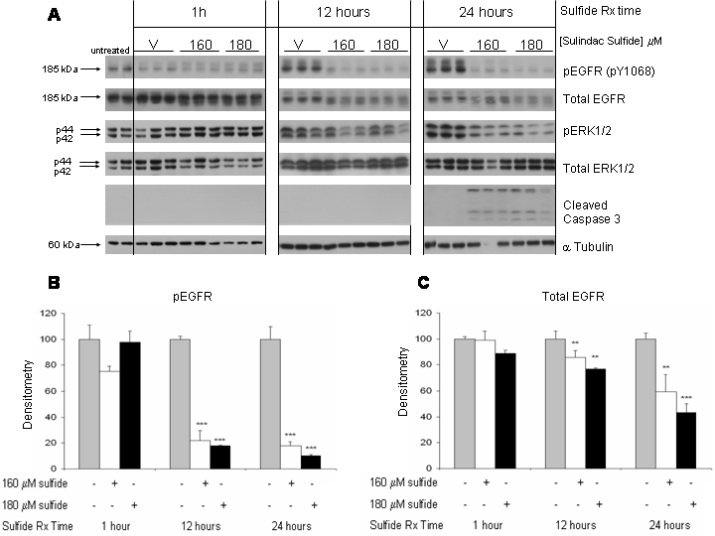
**Dose response and time course of sulindac sulfide inhibition of EGFR**. HT29 cells were grown to confluence in medium containing 10% FBS and treated with vehicle (0.1% DMSO), 160, or 180 μM sulindac sulfide for 1 h, 12 h, and 24 h. Cells were then harvested and immunoblots were performed on cell lysates with antibodies raised against pEGFR (pY1068), total EGFR, pERK1/2, total ERK1/2, and cleaved caspase-3; α-tubulin immunoblots of the same lysates served as loading controls. **(A) **1 h, 12 h, and 24 h immunoblot results. The graphs show the densitometry results of the pEGFR bands **(B) **and total EGFR bands **(C)**. Data represent mean of triplicate samples ± SD; statistical significance is denoted by **p < 0.01 and ***p < 0.001 *versus *respective time point vehicle. Results shown in figure are representative of 2 separate experiments each with triplicate samples.

Next we examined the time course of sulindac's effects on EGFR expression and phosphorylation in cells grown in 10% FBS (basal conditions). Phosphorylation of EGFR and total EGFR protein expression were downregulated as early as 12 h and persisted at 24 h following sulindac sulfide treatment (Figure 5). Significant downregulation of pERK1/2 was not detected prior to 24 h in these experiments. Evidence of apoptosis, by presence of caspase-3 cleavage products (Figure 5A) was also first detected at 24 h; examination of nuclear morphology after staining with acridine orange and ethidium bromide at 48 h confirmed cleaved caspase-3 immunoblot results (data not shown). Sulindac sulfide treatment did not consistently affect levels of total ERK1/2 or α-tubulin proteins. For this experiment immunoblots for each time point were run on individual gels thus, only samples within a single time point, assayed for one particular protein, can be directly compared.

**Figure 6:**
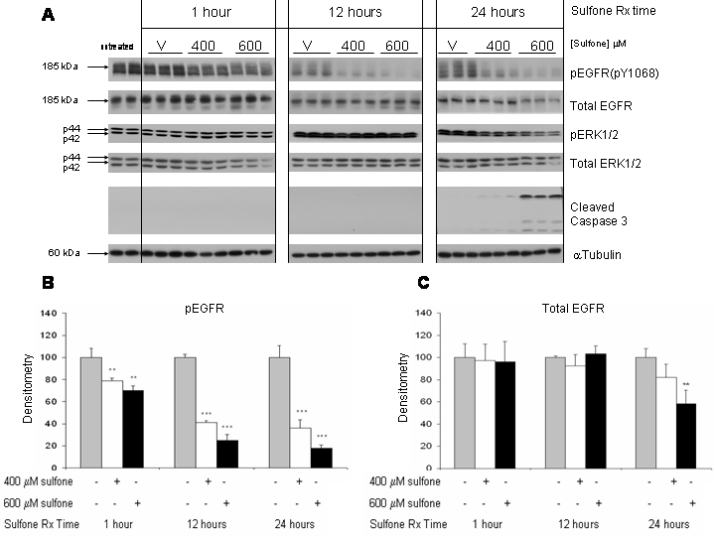
**Dose response and time course of sulindac sulfone inhibition of EGFR**. HT29 cells were grown to confluence in medium containing 10% FBS followed by treatment with vehicle (0.2% DMSO), 400, or 600 μM sulindac sulfone for 1 h, 12 h, and 24 h. Cells were then harvested and immunoblots were performed on cell lysates with antibodies raised against pEGFR (pY1068), total EGFR, pERK1/2, total ERK1/2, and cleaved caspase-3; α-tubulin immunoblots of the same lysates served as loading controls. **(A) **1 h, 12 h, and 24 h Western blot results. The graphs show the densitometry results of the pEGFR bands **(B) **and total EGFR bands **(C)**. Data represent mean of triplicate samples ± SD; statistical significance is denoted by **p < 0.01 and ***p < 0.001 *versus *respective time point vehicle. Results shown in figure are representative of 2 separate experiments each with triplicate samples.

The effect of sulindac sulfone on basal EGFR activation and expression was also examined. Sulindac sulfone treatment resulted in a modest but significant decrease in EGFR phosphorylation as early as 1 h after treatment, and this effect increased and persisted through 24 h (Figure 6A and 6B). Downregulation of total EGFR (Figure 6A and 6C) and pERK1/2 (Figure 6A) proteins was first seen at 24 h; caspase-3 cleavage (Figure 6A) was also first detected at 24 h. Morphologic evidence of apoptosis was apparent at 48 h, the earliest time point examined in this manner. Sulfone treatment did not consistently affect levels of total ERK1/2 or α-tubulin proteins.

To exclude the possibility that downregulation of EGFR by sulindac metabolites was unique to HT29 cells, similar experiments were carried out in Caco-2 and HCT116 cells. Sulindac sulfide and sulfone downregulated expression of both pEGFR and total EGFR in these cell lines as well (data not shown).

### ZVAD blocks sulindac-induced apoptosis but not EGFR downregulation

**Figure 7:**
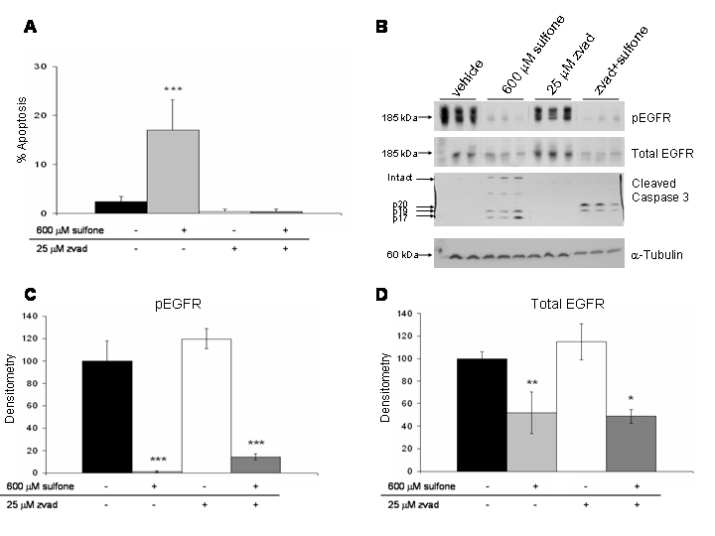
**Effect of the caspase inhibitor, ZVAD, on apoptosis and inhibition of EGFR**. HT29 colon cancer cells were grown to confluence in medium containing 10% FBS followed by pretreatment with or without 25 μM zvad for 1 h. Cells were then treated with vehicle (0.2% DMSO) or 600 μM sulfone for 48 h. Cells were harvested and immunoblots were performed on cell lysates with antibodies raised against pEGFR (pY1068), total EGFR, and cleaved caspase 3; α-tubulin immunoblots of the same lysates served as loading controls. The graphs show morphological apoptosis results **(A) **48 h Western immunoblot results **(B) **and densitometry of the pEGFR bands **(C) **and total EGFR bands **(D)**. Data represent mean of triplicate samples ± SD; statistical significance is denoted by *p < 0.05, **p < 0.01 and ***p < 0.001. Results shown in figure are representative of 2 separate experiments each with triplicate samples.

Although the downregulation of EGFR observed with sulindac metabolites occurred prior to either biochemical or morphologic evidence of apoptosis, it remains possible that some of the decrease in EGFR protein expression could be a consequence of caspase activation by sulindac metabolites. To examine this possibility, HT29 cells were pretreated with vehicle or 25 μM of the broad specificity caspase inhibitor ZVAD-fmk (ZVAD) for 60 minutes prior to treatment with 600 μM sulindac sulfone or vehicle for 48 h. ZVAD pretreatment completely blocked sulindac sulfone induced apoptosis as measured by examination of nuclear morphology (Figure 7A) and it prevented cleavage of caspase 3 to the active 17 and 19 kDa fragments (Figure 7B; the 20 kDa caspase 3 fragment seen with the combination of sulfone and ZVAD has been shown to be an inactive cleavage product [26-29]. While 25 uM ZVAD prevented both caspase activation and morphologic apoptosis induced by sulindac sulfone, it did not prevent sulindac sulfone induced decreases in either total or pEGFR (Figure 7B–D). Combination experiments with ZVAD and sulindac sulfide showed similar results as those shown in Figure 7 for sulindac sulfone. These results demonstrate that downregulation of EGFR by sulindac metabolites is not a consequence of the apoptotic effect of these drugs.

## Discussion

Our laboratory originally reported that sulindac metabolites inhibit the EGFR/mitogen activated protein kinase (MAPK)/ERK1/2 signaling pathway at the level of MEK or higher [5] (Figure 1) and that the downregulation of mitogen-activated protein kinase kinase (MEK)/ERK1/2 is both necessary and sufficient for the apoptotic effect of these drugs [8]. We now report, for the first time, that sulindac metabolites inhibit EGFR phosphorylation and downregulate the expression of total EGFR protein in human colon cancer cells. Downregulation of EGFR occurred at doses and within a time course that is consistent with the possibility that the effect of drug on the EGFR could be the mechanism of the inhibition of MEK1/2 and ERK1/2 activity and apoptotic cell death induced by these drugs.

Both sulindac sulfide and sulindac sulfone downregulate EGFR expression and phosphorylation at comparable doses to those that inhibit ERK1/2 phosphorylation and induce apoptotic cell death (Figures 5 and 6). The time course experiments suggest that the kinetics of the EGFR effect may differ between the two sulindac metabolites. With sulindac sulfide, the effect on EGFR and ERK1/2 occurred concurrently (first seen at 12 h) and before any detectable effect on apoptosis. This suggests that the downregulation of EGFR may be responsible for some or all of the ERK1/2 inhibitory effects of the drug. The relationship between sulindac sulfone's effect on EGFR and ERK1/2 is not as clear since an effect on pEGFR was detected substantially earlier (1 hour) than the effect on pERK1/2 and apoptosis (24 hours). These differences in the kinetics of the two sulindac metabolites on pEGFR and pERK1/2 downregulation could represent differences in potency, duration of effect, the spectrum of EGFR phosphorylation sites that are affected by the different drugs, differential effects on phosphatases, and/or differences in the molecular mechanism of action. Similar to our results, Jimeno et al. reported that treatment of HuCCT cells with the EGFR tyrosine kinase inhibitor (TKI) erlotinib or the mononclonal antibody (MAb) cetuximab led to significant downregulation of pEGFR as early as 1 h following treatment whereas major downstream effectors (pERK1/2 and pAKT) were not coordinately affected [18]. Despite these differences in kinetics, the finding that EGFR downregulation was observed consistently with both sulindac metabolites and in three different CRC cell lines (HT29, HCT116, and Caco-2) establishes the EGFR as a new target of sulindac.

Activation of the EGFR pathway is particularly common in colorectal cancers [19-22] and overexpression of EGFR correlates with more aggressive disease and poor prognosis [23-26]. Both EGFR MAbs and TKIs that block activation of EGFR are now being used for the treatment of colorectal cancer [27]. In light of our evidence that NSAIDs downregulate EGFR signaling and induce apoptosis, it is interesting to note that both EGFR MAbs and TKIs have been shown to stimulate important pro-apoptotic genes and downregulate anti-apoptotic genes, particularly those of the Bcl-2 family [28-30]. Furthermore, treatment with combinations of agents that inhibit EGFR by different mechanisms is more effective than treatment with a single agent. For example, the combination of erlotinib plus cetuximab caused a synergistic induction of apoptosis *in vitro*, and an additive effect on tumor growth arrest *in vivo *[18]. Thus, downregulation of EGFR could mediate the apoptotic and chemopreventive effects of NSAIDs and could explain some of the clinical evidence of additive growth inhibitory effects of EGFR inhibitors and NSAIDs. These data suggest a biochemical rationale for the use of combinations of classic EGFR inhibitors and NSAIDs in the treatment or prevention of CRC.

If inhibition of EGFR signaling is a mechanism of NSAIDs as our data indicates, it will be of great interest to examine the full spectrum of possible consequences of downregulation of EGFR. Thus far, we have only examined the ras-raf-MEK1/2-ERK1/2 arm of EGFR signaling but several other signaling pathways are known to be activated by the EGFR including the phosphatidylinositol-3-kinase (PI3-K) and Janus kinase (JAK)-signal transducers and activators of transcription (STAT) pathways [31]. Sulindac metabolites may also affect other members of the ErbB family of receptor tyrosine kinases (RTK) including Erb2, ErbB3, ErbB4, non-ErbB RTKs including PDGFR and VEGFR, and/or receptors lacking tyrosine kinase activity such as the adenosine receptor. Studies to examine the effect of NSAIDs on these pathways and receptors are underway.

We have not systematically examined the mechanism by which sulindac metabolites downregulate EGFR but the relatively short time course (as early as 1 hour) suggests that the effect is more likely due to increased degradation of EGFR rather than inhibition of new protein synthesis. Our data demonstrate that EGFR inhibition is not a consequence of caspase activation or due to the apoptotic effects of the drugs in that apoptosis was first detected 24 h after sulindac metabolite treatment, whereas downregulation of EGFR was seen as early as 1 to 12 hours after drug treatment. In addition, inhibition of apoptosis with the broad specificity caspase inhibitor ZVAD did not prevent EGFR downregulation by sulindac metabolites. Thus, we hypothesize that EGFR downregulation by sulindac metabolites is not due to activation of caspases, but rather is a result of increased proteasomal and/or lysosomal degradation. Further studies are warranted to determine the precise mechanism of EGFR downregulation by sulindac metabolites.

Sulindac metabolites decreased expression of both total and pEGFR proteins. The time course experiments, particularly those with sulindac sulfone (Figure 6), suggest that downregulation of these two forms of the receptor could be occurring by distinct mechanisms. Although total and pEGFR downregulation with sulindac sulfide occurred concurrently, the sulfone metabolite decreased pEGFR as early as 1 h whereas downregulation of total EGFR protein was first observed at the 24 h time point. This sequence indicates that the downregulation of pEGFR is not likely to be a consequence of the downregulation of the total receptor, at least with sulindac sulfone.

## Conclusion

In summary, we have described for the first time that sulindac metabolites downregulate EGFR and prevent EGFR phosphorylation by EGF. This effect may explain, at least in part, our previous observation that these drugs inhibit basal and EGF-induced activation of the MEK/ERK arm of the EGFR signaling pathway. Downregulation of EGFR was shown to be independent of COX inhibitory activity as it occurred with sulindac sulfone. These results add to the increasing amount of data that suggest interactions exist between the effects of NSAIDs and EGFR signaling [13,14]. Our results are the first to suggest that these interactions are due to a direct drug effect on the EGFR itself.

## Abbreviations

NSAID, non-steroidal anti-inflammatory drug; CRC, colorectal cancer; ERK1/2, extracellular-signal regulated kinase; EGF, epidermal growth factor; EGFR, epidermal growth factor receptor; pEGFR, phosphorylated epidermal growth factor receptor; pERK1/2, phosphorylated extracellular-signal regulated kinase 1/2; COX, cyclooxygenase; FAP, familial adenomatous polyposis; FBS, fetal bovine serum; PBS, phosphate buffered saline; MAPK, mitogen-activated protein kinase; MEK1/2, mitogen-activated protein kinase kinase; MAb, monoclonal antibody; TKI, tyrosine kinase inhibitor; PI3-K, phosphatidylinositol-3-kinase; JAK/STAT, Janus kinase/signal transducers and activators of transcription; RTK, receptor tyrosine kinase
